# Cholecystokinin-Like Peptide (DSK) in *Drosophila*, Not Only for Satiety Signaling

**DOI:** 10.3389/fendo.2014.00219

**Published:** 2014-12-18

**Authors:** Dick R. Nässel, Michael J. Williams

**Affiliations:** ^1^Department of Zoology, Stockholm University, Stockholm, Sweden; ^2^Functional Pharmacology, Department of Neuroscience, Uppsala University, Uppsala, Sweden

**Keywords:** neuropeptide, peptide hormone, aggression, feeding, intestinal function, locomotion

## Abstract

Cholecystokinin (CCK) signaling appears well conserved over evolution. In *Drosophila*, the CCK-like sulfakinins (DSKs) regulate aspects of gut function, satiety and food ingestion, hyperactivity and aggression, as well as escape-related locomotion and synaptic plasticity during neuromuscular junction development. Activity in the DSK-producing neurons is regulated by octopamine. We discuss mechanisms behind CCK function in satiety, aggression, and locomotion in some detail and highlight similarities to mammalian CCK signaling.

## Introduction

Many neuropeptide signaling pathways are well conserved over evolution (1–3). One example is the cholecystokinin (CCK) signaling that regulates satiety and food intake in nematode worms, insects, and mammals (4–8). The first CCK-like peptide of insects was isolated from the cockroach, *Leucophaea maderae*, and designated leucosulfakinin (9). In *Drosophila*, two sulfakinins (DSK1 and DSK2; encoded by CG18090) and two DSK receptors (CCKLR1 and CCKLR2; encoded by CG6881 and CG6857) have been identified (10–12). The DSKs display strong sequence similarities to vertebrate gastrin/CCKs and sulfakinins (SKs) of other invertebrates (Table 1) and also their G-protein coupled receptors (GPCRs) are well conserved, suggesting a ligand–receptor coevolution (13). Actually, the amino acid sequences of the two DSKs are identical in the nine residues of the carboxy terminus, and DSK-II is N-terminally extended by five residues compared to DSK-I (12) (Table 1). The tyrosine residues of mammalian CCK, and many of the insect sulfakinins, are sulfated, a modification essential for proper activation of their receptors.

**Table 1:** **Sequences of CCK-like peptides^a^**.

DSK-I	*Drosophila melanogaster*	FDD**Y**GHMRFamide
DSK-II	*Drosophila melanogaster*	GGDDQFDD**Y**GHMRFamide
DSK 0	*Drosophila melanogaster*	NQKTMSFamide
LSK	*Leucophaea maderae*	pQSDD**Y**SGHMRFamide
CCK-1	*Aplysia californica*	SYGDYGIGGGRFamide
CCK-2	*Aplysia californica*	QGAWSYDYGLGGGRFamide
NPL-12a	*Caenorhabditis elegans*	DYRPLQFamide
NPL-12b	*Caenorhabditis elegans*	DGYRPLQFamide
CCK8	Mammals	D**Y**MGWMDFamide

*^a^Sequences from Ref. (2, 6, 9, 12). Bold tyrosine (Y) residues are sulfated*.

In mammals, CCK secreted from the intestine acts on receptors in the nucleus of the solitary tract of the brain to signal satiety and thus inhibit feeding (7, 14). In *Drosophila*, DSKs released from insulin-producing cells (IPCs) of the brain appear sufficient to induce satiety (8, 15). As with many neuropeptides and peptide hormones, CCK is multifunctional and can also act locally in the intestine to decrease gastrointestinal motility, stimulate secretion of pepsinogen, inhibit gastric acid secretion, stimulate gallbladder contraction, and trigger secretion of hormones in the pancreas (13, 16). Furthermore, CCK released from brain neuroendocrine cells has regulatory functions in nociception, memory and learning processes, panic, and anxiety (17). To what extent are these CCK functions subserved by DSKs in *Drosophila* and SKs of other insects? We provide a brief update on DSK signaling in *Drosophila* and show that, in addition to inducing satiety, several functions are conserved over evolution.

KEY CONCEPT 1. Neuropeptides and peptide hormones Neuropeptides and peptide hormones typically consist of 5–80 amino acids linked by peptide bonds. They act on G-protein-coupled receptors (GPCRs) or in some cases receptor tyrosine kinases. In Drosophila, about 50 genes have been identified that encode precursors of neuropeptides or peptide hormones, and more than 45 GPCRs are known.

KEY CONCEPT 2. Neuroendocrine cells Neuropeptides and peptide hormones are produced by a variety of neurons, and neuroendocrine cells in the central and peripheral nervous system as well as in glandular cells in other tissues, including the intestinal tract. Within the CNS, peptidergic neurons form a large variety of modulatory circuits. Peptide hormones are typically released into the circulation.

## Multiple Roles of Sulfakinins in Intestinal Function in Insects

In several insects, it was shown that SKs modulate spontaneous activity of the foregut and hindgut muscle, as well as heart contractions [see Ref. (3, 9, 13, 18)]. In some insect species, SKs were also shown to induce secretion of the digestive enzyme α-amylase (19). In *Drosophila*, the enteroendocrine cells of the gut do not produce DSKs, and there is no direct innervation of the posterior intestine by DSK-expressing neurons (20, 21). Thus, actions of DSKs on the more posterior intestine are likely to be hormonal, via release from median neurosecretory cells (MNCs) that also produce insulin-like peptides and have axon terminations in neurohemal regions of the corpora cardiaca and anterior aorta, crop duct, and anterior midgut (8, 22). The DSK action on the heart, crop, and anterior intestine could, however, be by means of direct release onto these structures by axon terminations of the same neurosecretory cells. Taken together, these findings indicate that insect SKs and mammalian CCKs have some conserved actions in relation to intestinal function.

KEY CONCEPT 3. Median neurosecretory cells In insects, a region in the dorsal midline of the brain, designated pars intercerebralis, contains a group of median neurosecretory cells (MNCs) that produce a number of peptide hormones, including insulin-like peptides (DILPs) and CCK-like peptides (DSKs). This brain region is considered functionally analogous to the hypothalamus.

## Satiety Signaling

Endocrinological studies demonstrated that SKs inhibit food intake in several insects, such as blowfly, locust, cricket, cockroach, and the beetle *Tribolium castaneum* (5, 13, 23–26). In *Drosophila*, targeted genetic interference with expression of DSKs also revealed that these peptides are important for satiety signaling (8, 15). In the adult *Drosophila* CNS, there is a small number of DSK-producing neurons: four very large interneurons posteriorly in the brain, about eight smaller interneurons dorsolaterally and ventrally in the brain, and a varying number of MNCs (8, 20). The DSK-expressing MNCs are a subpopulation of the 14 IPCs (8). Thus, most of the IPCs produce both the insulin-like peptides DILP2, 3, and 5 and the DSKs.

KEY CONCEPT 4. Satiety signaling Hunger and satiety can be determined either by willingness to approach food, or by amount of food ingested. Food intake in adult Drosophila can be monitored by a capillary feeding (CAFE) assay: flies feed from a calibrated capillary, and the amount ingested is calculated from the diminished level in the capillary. In some cases colored food is used and ingestion is determined by spectrophotometry.

Knockdown of DSK by RNAi targeted to DSK-producing neurons decreased satiety signaling in flies and hence intake of food increased, even when it was less palatable with no sugar or bitter with caffeine added (8). It was furthermore shown that knockdown of DSK only in the IPCs was sufficient to produce the same phenotype, suggesting that the hormonal action of DSKs is important as a satiety signal. Similar results were obtained from third instar larvae. In flies, inactivation of the IPCs or all the DSK-producing neurons by targeted expression of a hyperpolarizing potassium channel (Ork1) generated the same phenotype on food intake, indicating that activity in the IPCs is required to induce satiety (8). Flies deficient in DSKs displayed increased resistance to starvation compared to control flies, probably as a consequence of the dysregulated satiety signaling and resulting increase in food intake (8). Interestingly, knocking down DSKs either in IPCs or in all DSK-producing cells led to compensatory increases of *Dilp2, 3*, and *5* transcripts in the brain of flies fed *ad libitum*, but had no effect on flies starved for 24 h. Another study revealed that the *Drosophila* obesity-linked homologs Transcription factor AP-2 (TfAP-2) and Tiwaz (Twz) regulate octopamine signaling to initiate feeding and then octopamine, in a negative feedback loop, induces expression of *Dsk* to inhibit consummatory behavior (15). Combined, these findings suggest that DSKs released from IPCs are sufficient to induce satiety in larval and adult *Drosophila*, but the mechanisms remain elusive. The DSK receptor localization and targets of the peptide are yet to be identified, and it remains possible that the action could be either central or peripheral.

**Figure 1:**
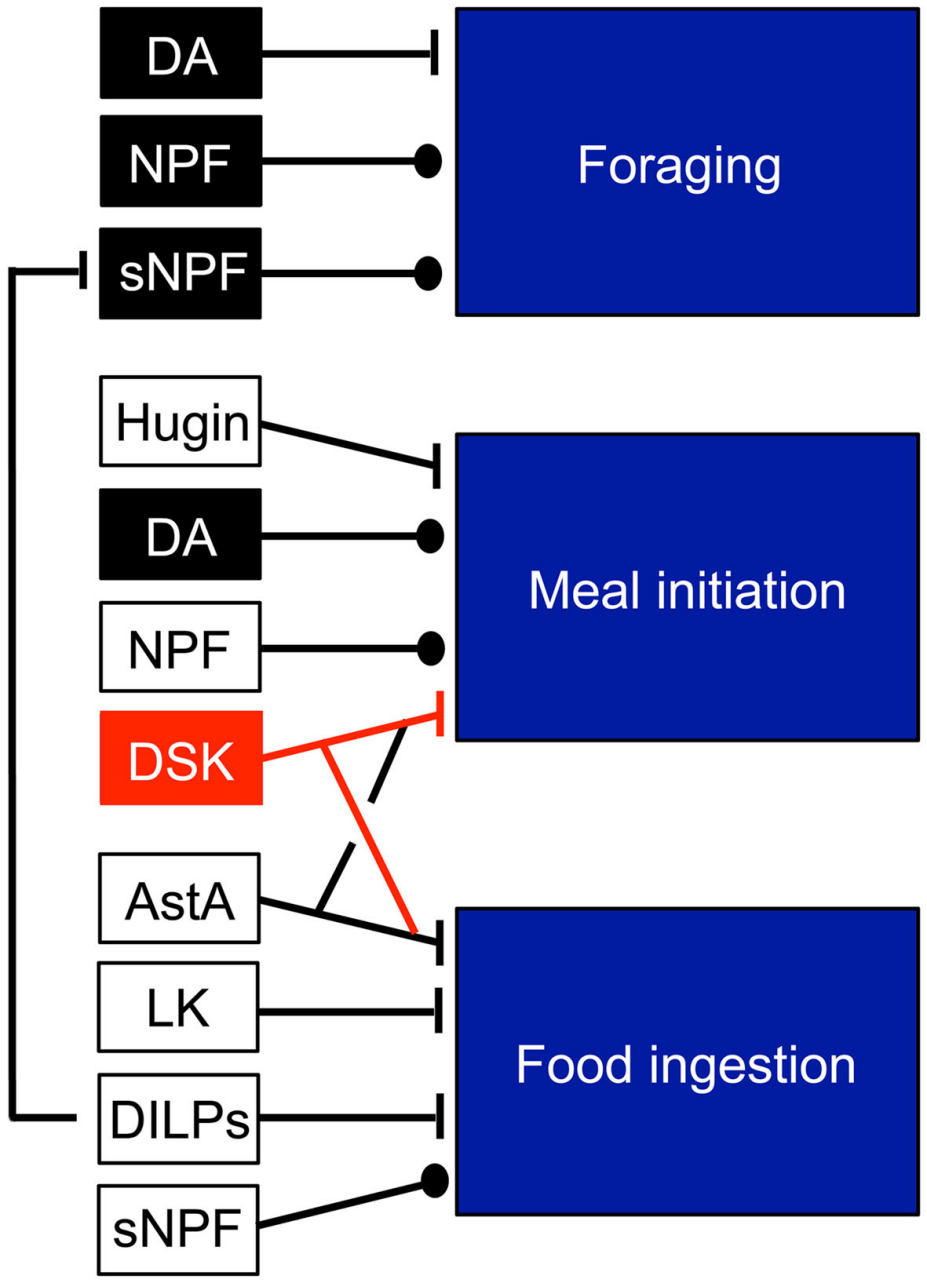
**Neuromodulators that regulate feeding modules in *Drosophila***. The neuromodulators in black and red units are produced by identified neurons: dopamine (DA) in DL1 neurons (for foraging) and in ventral unpaired neurons (for meal initiation), NPF in non-clock NPF neurons, sNPF in olfactory sensory neurons (OSNs), DSK in IPCs. The other peptides Hugin, allatostatin A (AstA), leucokinin (LK) are produced by several neurons types and it is not known which mediate the feeding responses. Probably, DILPs from IPCs contribute to inhibition of food ingestion. Note that DILPs inhibit the sNPF signaling in OSNs. This figure was compiled from data in Figure 3 in Ref. (29).

There are several sets of neurons in the brain known to regulate feeding. Among these are the so-called hugin neurons that produce a neuropeptide of pyrokinin type, whose branches are known to superimpose those of the IPCs (27). Functional interactions between the brain IPCs and the hugin neurons were demonstrated recently (28). The IPCs could signal to the hugin neurons by both DILPs and DSKs and thereby regulate the activity in these neurons that are at the interface between gustatory inputs and feeding regulation. There are several other candidate targets among central neurons. Neurons in circuits that use the following neurotransmitters and neuropeptides have been implicated in the regulation of foraging and feeding in addition to DILPs and DSKs: dopamine (DA), neuropeptide F, short neuropeptide F, allatostatin A, leucokinin, and hugin [see Ref. (3, 29)]. These sets of neurons are shown schematically in Figure 1.

## Aggression and Anxiety

It was demonstrated that CCK signaling through the CCK B receptor (CCKBR) within the rodent brain induces hyperactivity and aggression (30, 31). In support of this, CCKergic neuronal projections were identified within the limbic system, the brainstem, and the cerebral cortex, many of which overlap with neuronal pathways considered to be significant for the modulation of fear, anxiety, and aggression [for review, see Ref. (32)]. Furthermore, overexpression of CCKBR in the mouse brain increased aggressive behavior, while mice lacking CCKBR displayed increased exploratory behavior and reduced anxiety (31, 33).

**Figure 2:**
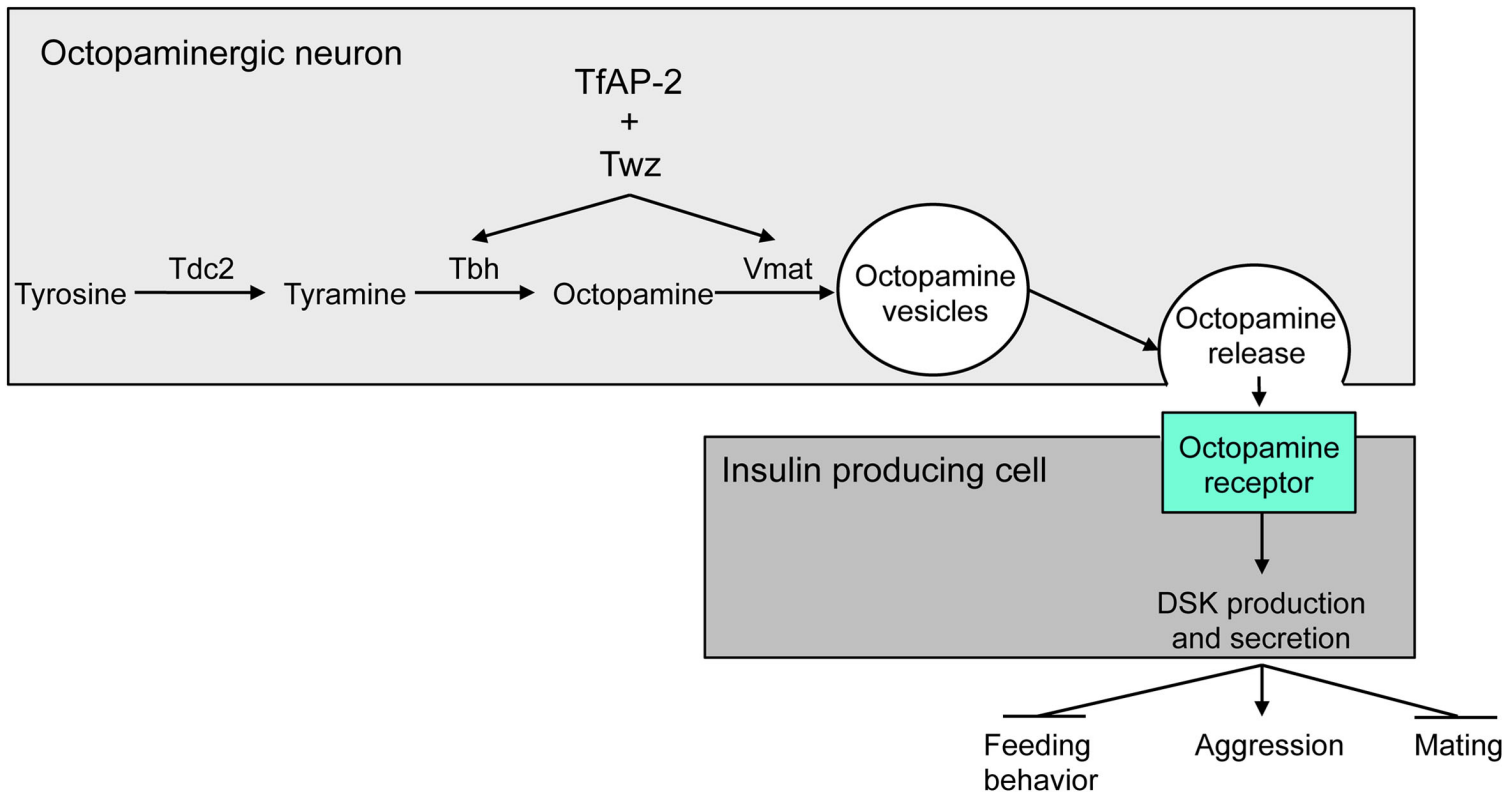
**Model for the regulation of aggressive behavior by DSK**. Twz and TfAP-2 regulate the expression of Tbh and Vmat, which control octopamine production and release from octopaminergic neurons. Octopamine then signals to the IPCs to induce *Dsk* transcription. *Dsk* signals to stimulate aggression, while inhibiting mating and feeding behavior (15). This figure was modified from Figure 8 in Ref. (36).

In *Drosophila*, while DA and serotonin are involved in the modulation of aggressive behavior, the central regulator of aggression is the noradrenaline analog, octopamine (34–37). Recently, it was reported that *Drosophila* homologs of the human obesity-linked genes *TFAP2B* and *KCTD15* [*TfAP-2* and *Tiwaz* (*Twz*)] regulate at least two genes involved in the production and secretion of octopamine within the brain, *Tyramine* β *hydroxylase* (*Tbh*) and *Vesicular monoamine transporter* (*Vmat*) (Figure 2). Octopamine then regulates aggression, mating, and activity in *Drosophila* by controlling the expression of Dsk in the IPCs (36) (Figure 2).

KEY CONCEPT 5. Aggressive behavior Male fruitflies fight over mates and resources and display elaborate aggressive behavior. The neuronal circuitry regulating aggressive behavior in Drosophila is intensely studied with genetic methods. Both monoamines and neuropeptides are known modulators of aggression in flies. It has been possible to identify small groups of neurons that regulate specific aspects of aggression.

Overexpressing TfAP-2 in octopaminergic neurons was sufficient to induce the expression of *Dsk*. This *Dsk* induction was blocked by feeding males an octopamine antagonist, indicating that TfAP-2 and Twz induce *Dsk* expression via octopamine signaling (Figure 2). Furthermore, *Dsk* overexpression in the IPCs was itself sufficient to induce hyperactivity and aggressive behavior. Interestingly, TfAP-2-induced aggressive behavior was blocked by feeding flies, a CCK antagonist. This suggests that octopamine-induced aggression is due to an increase in DSK signaling (Figure 2).

## Development of the Neuromuscular Junction and Modulation of Locomotion

Similar to mammals, the *Drosophila* genome encodes two different Dsk receptors, CCKLR1 (CCKLR-17D1) and CCKLR2 (CCKLR-17D3). In *Drosophila* larvae, CCKLR-17D1 signaling was reported to be necessary for body-wall muscle contractions involved in stress-induced escape behavior (38). Moreover, it was demonstrated that *Dsk* and CCKLR-17D1 are required for proper neuromuscular junction (NMJ) formation in larvae (39). Interestingly, another study reported that octopamine regulates synaptic plasticity in the NMJ during development, as well as under starvation conditions. By activating Octβ2R receptors in octopaminergic neurons, octopamine initiates signaling events that induce the development of new synaptic boutons at larval NMJs (40, 41). This lends itself to the hypothesis that, similar to what was reported in the *Drosophila* brain, octopamine and *Dsk* interact at NMJs to regulate their development, as well as plasticity under condition of increased locomotor behavior.

## Conclusion and Outlook

The CCK-like peptides, DSKs, of *Drosophila* and SKs of other insects regulate gut function, satiety/food ingestion, hyperactivity, and aggression, as well as escape-related locomotion and synaptic plasticity during NMJ development. Thus, many of the functional roles of CCK signaling known in mammals are present also in insects. Recent studies have shown that the neurons producing DSKs are under regulatory control by octopaminergic neurons (36) and more specifically the IPCs that co-express DILPs and DSKs are regulated by the octopamine receptor OAMB (42). An important question for the future is to determine the targets of DSK signaling within the brain and at peripheral sites that regulate the different aspects of behavior and physiology.

## Conflict of Interest Statement

The authors declare that the research was conducted in the absence of any commercial or financial relationships that could be construed as a potential conflict of interest.
